# High iron-mediated increased oral fungal burden, oral-to-gut transmission, and changes to pathogenicity of *Candida albicans* in oropharyngeal candidiasis

**DOI:** 10.1080/20002297.2022.2044110

**Published:** 2022-03-01

**Authors:** Aparna Tripathi, Anubhav Nahar, Rishabh Sharma, Trevor Kanaskie, Nezar Al-Hebshi, Sumant Puri

**Affiliations:** Oral Microbiome Research Laboratory, Department of Oral Health Sciences, Kornberg School of Dentistry, Temple University, Philadelphia, Pennsylvania, USA

**Keywords:** Candida albicans, oropharyngeal candidiasis, iron, antifungal-resistance, cell wall, β-1, 3-glucan, phagocytosis

## Abstract

**Background:**

Iron affects the diversity of the oral microbial landscape. Laboratory-strain CAI4 of *Candida albicans* that causes oropharyngeal candidiasis (OPC) exhibits iron-induced changes to the cell wall, impacting phagocytosis (by macrophages) and susceptibility of fungal cells to cell wall-perturbing antifungals, *in vitro*.

**Aim:**

To understand the effect of iron on the CAI4-strain, wild type (WT) SC5314-strain, and oral isolates of *C. albicans*.

**Methods:**

An immunosuppressed murine model of OPC was used to assess the effect of iron on oral-to-gut infection and antifungal susceptibility of the CAI4-strain. *In vitro* antifungal susceptibility, cell wall analysis, and phagocytic assays were performed under low and high iron, for the SC5314-strain and oral isolates.

**Results:**

High iron enhanced oral and gut fungal levels for the CAI4-strain in mice; CAI4 cells from low iron mice were more susceptible to antifungals. The SC5314-strain and oral isolates showed enhanced antifungal-resistance towards most antifungals tested, under high iron. Iron-mediated cell wall changes and phagocytic response in the SC5315-strain were similar to CAI4; oral isolates showed a variable response.

**Conclusion:**

Host iron can potentially alter infection severity and dissemination, efficacy of antifungal treatment, and host immune response during OPC. Clinical isolates showed most of these effects of iron, despite exhibiting a varied cell wall composition-change response to iron.

## Introduction

*Candida albicans* is the most common commensal fungus in healthy individuals, and the primary causative agent of oropharyngeal candidiasis (OPC) in the immunocompromised [1]. Changes in the host-specific environment, including alterations in iron levels, can cause variations in the *C. albicans* burden and affect its virulence 2). Iron is an essential nutrient that can be sourced by oral microbes from inorganic iron (in saliva), heme-bound iron (in crevicular fluid and non-vegetarian diet), and non-heme iron (in vegetarian diet); or can be sequestered from iron-binding proteins like transferrin, lactoferrin, and hemoglobin. Lack of a divalent metal transporter in the oral epithelium, host-related factors like iron-rich diet, and abundance of acid-producing bacteria that increases iron bio-availability by reducing environmental pH [3], can potentially make the oral environment particularly iron-rich.

In order to sustain its growth in the oral cavity, *C. albicans* can obtain iron from multiple host sources (except lactoferrin) [4]. Treatment with the FDA-approved oral iron chelator deferasirox (DFX) reduced the *C. albicans* burden during OPC in mice (Puri et al. 2019b). This raises the possibility that an increase in host iron levels can enhance the fungal load in the oral cavity. Oral microbes are continuously ingested and cause gut microbial dysbiosis [5]. Therefore, a high iron-mediated increase in the oral fungal load can potentially facilitate enhanced *Candida* gut colonization, which is a source of life-threatening disseminated candidiasis [6].

The fungal cell wall plays a crucial role in the virulence of systemic and mucosal infections, including OPC. The dynamic structure of the *C. albicans* cell wall is crucial for cell shape, viability, pathogenesis, interaction with other microbial species, and evasion of host immune cells [7]. The outer layer of the cell wall is made of mannans, while the inner layer consists of β-1,3-glucan complexed with chitin, along with small amounts of β-1,6-glucan [8]. Mannans mask β-1,3-glucans and a decrease in mannan levels induces β-1,3-glucan exposure on the cell surface. Also, an increase in chitin levels can be associated with enhanced exposure of β-1,3-glucan [9,10]. As the primary fungal pathogen-associated molecular pattern (PAMP), β-1,3-glucan binds to Dectin-1, a C-type lectin pattern recognition receptor (PRR) of the host’s immune cells [11]; and promotes phagocytosis and inflammatory responses [12].

Factors such as environmental pH, zinc, iron, and lactic acid can alter the β-1,3-glucan exposure as well as the actual levels of mannan, β-1,3-glucan, or chitin in wild-type (WT) and common laboratory strains of *C. albicans* [13–16]. We have recently shown that a commonly used laboratory strain of *C. albicans* (CAI4 that is prototrophic for uracil biosynthesis, Ura^+^) modulates its cell wall in response to environmental iron, altering its susceptibility to cell wall-perturbing (CWP) agents and host immune response [16]. An iron-rich environment enhanced β-1,3-glucan exposure-dependent phagocytosis [16], while cell wall structural changes also affected the response to antifungals including CWP agents [13,16]. However, strain heterogeneity among clinical isolates of fungi can affect virulence traits [17]. This underscores the need to evaluate such isolates to understand how they may differ from the WT and laboratory strains of their respective species. The effect of iron on the cell wall architecture, drug susceptibility, and immune recognition of oral clinical isolates of *C. albicans* that have already been exposed to different oral challenges in the host is completely unknown.

Here, we analyze the effect of high iron on oral infection and oral-to-gut transmission, along with the effect of iron on drug resistance, during OPC. In addition, we evaluated the effect of iron on changes in the fungal cell wall and the resulting modulation in antifungal-sensitivity and macrophage-mediated phagocytosis, for both WT and oral isolates of *C. albicans*.

## Material and methods

### Fungal strains, media, culture conditions, and animals

*C. albicans* CAI4 (Ura^+^) strain (Δura3::imm434/Δura3::imm434RPS1/Δrps1::Clp10-URA3) 2 and WT SC5314 strain (ATCC, MYA-2876™) were used, along with *C. albicans* oral isolates. These clinical isolates were collected over the past 2 years from the dental clinics at the Temple University, and strain identification was performed at the Oral Microbiome Research Laboratory, initially using Chrom Agar^TM^ (BD), followed by molecular characterization, as described in the Supplemental section (Figure S2 and Supplemental methods). Isolates from de-identified yeast extract-peptone-dextrose (YPD; Difco) plates were regrown in YPD and stored at −80°C as glycerol stocks. *C. albicans* exponential-phase cells were prepared as described previously [16] in yeast nitrogen base (YNB; 4027–112; MP Biomedicals) limited iron medium as low iron medium or the same with the addition of 50 µM bathophenanthrolinedisulfonic acid (iron-chelator) and 100 µM FeCl_3_.6H_2_O as replete iron medium (or high iron medium) [2]. For antifungal sensitivity assays, uridine (50 μg ml^−1^) containing YNB or YPD agar plates were used. For experiments involving animals, 4 to 6 weeks old C57BL/6 female mice from Jackson Labs, Bar Harbor, ME, were used.

### *Murine model of OPC and tissue collection for* C. albicans *Cfu determination*

The immunosuppression model of murine OPC was used, as described previously [18], with the following modifications: 1) Mice were divided into two groups: control (without treatment; n = 8) and iron-overload (treated with 10 mg/kg of body weight of iron-dextran (Sigma) intraperitoneally [19] at day −1; n = 8); 2) Tranquilizer chlorpromazine hydrochloride (10 mg kg^−1^) was given to each mouse on day 0, before infection [20]. On day 5, the mice were euthanized; and tongue, stomach (upper half of the body including 5–7 mm of the esophagus), and small intestine (10 cm in length, recovered from the cecum side) were excised. The right lateral half of each mouse's tongue and whole samples of the stomach and small intestine were weighed and homogenized in PBS. Serial dilutions of tissue mixture of all mice were plated individually on YPD-agar plates (containing 50 μg ml^−1^ uridine and streptomycin*/*penicillin) and incubated at 30^ᵒ^C for 48 h. The *C. albicans* burden in tissues was assessed by Cfu (colony forming unit) quantification.

### Antifungal sensitivity

#### Sensitivity of C. albicans grown under low and high iron in vivo

To assess the antifungal sensitivity of *C. albicans* grown *in vivo* under low and high iron conditions, we used our immunosuppressed murine model of OPC, as described above. The mice were divided into two groups: 1) high iron mice (untreated) and 2) low iron mice (treated with DFX). The DFX mice group was treated by prophylactic iron chelation therapy with 10 mg kg^−1^ DFX (Novartis) in suspension of sodium chloride solution (0.9% w/v) using oral gavage [21]. A total of 8 mice per group were included. On day 5, homogenized tissue mixtures were prepared as described in the above section and pooled together per group. Pooled tissue mixtures of DFX-treated and untreated mice groups were plated on YPD-agar plates (as described above) with or without antifungals [1 μg ml^−1^ tunicamycin, 75 ng ml^−1^ caspofungin (Sigma-Aldrich), an inhibitor of β-1,3-glucan synthase enzyme, and 12.5 μg ml^−1^ calcofluor white (CFW)] and incubated at 30^ᵒ^C for 48 h. Sensitivity of *C. albicans* was assessed by Cfu-quantification. Plates without antifungals were considered as control for respective mice groups.

#### C. albicans sensitivity in vitro

*C. albicans* susceptibility to tunicamycin (1.75 μg ml^−1^) and CFW (100 μg ml^−1^) were tested by a spot dilution assay, while zymolyase sensitivity was assessed in liquid media by measuring *C. albicans* growth (O.D._600_) in the presence of 30 U zymolyase, for 4 h, as described previously [16].

#### Minimum inhibitory concentration (MIC)

For evaluation of clinical antifungal drug sensitivity towards *C. albicans* grown in low or high iron medium, exponential cells of *C. albicans* were diluted to an O.D._600_ of 0.1 in their respective medium and incubated at 30^ᵒ^C for 24 h in the absence (as control) or presence of drugs. The following antifungal drugs were tested: amphotericin B (0.125–16 μg ml^−1^), nystatin (0.5–16 μg ml^−1^), fluconazole (0.125–16 μg ml^−1^), and caspofungin (0.03–4 μg ml^−1^). The MICs of antifungal drugs were determined to have the lowest concentration that inhibited ≥90% of the *C. albicans* cells.

### Fluorescent staining and analysis of cell wall components

Concanavalin A (ConA), CFW, and aniline blue (AB) were used to stain *C. abicans* mannan, chitin, and β-1,3 glucan, respectively, while β-1,3 glucan exposure was detected by the anti-β-1,3 glucan antibody-based staining method, as described previously [16]. Images were taken with a confocal microscope at 100 × magnification and quantified by measuring mean fluorescence intensities (MFI) using the ImageJ software.

### Phagocytosis

The phagocytosis assay was followed by *C. albicans* co-incubation with primary murine bone marrow-derived macrophages (BMDM) at a multiplicity of infection (MOI) of 5:1 in 1 ml of RPMI-1640 (Hyclone) media for 3 h at 37°C under 5% CO_2_, as described previously [16].

### Ethics statement

All animal experiments were performed as per guidelines of the Animal Care and Use Protocol, under the protocol (ACUP: 4623) reviewed, and approved by the institutional Animal Care and Use Committee (IACUC) of the Temple University to ensure proper care and handling of the laboratory animals.

### Statistical analysis

Statistical analysis was performed using the Mann-Whitney or Student t-test, as indicated, between the low and high iron study groups, using the GraphPad Prism software version 7 (USA).

## Results

### *High host iron increases* C. albicans *oral burden and oral-to-gut dissemination*

**Figure 1.**
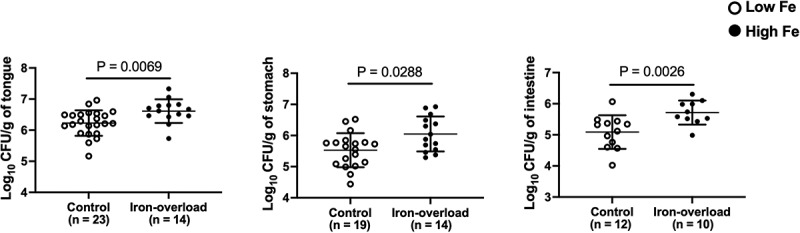
Iron-overload causes enhanced infection and promotes oral-to-gut dissemination of *C. albicans* during murine OPC. The numbers of Cfu per gram of tissue, tongue (a), stomach (b), and intestine (c), were obtained from *C. albicans* (CAI4, Ura^+^)-infected untreated (control) or iron-dextra-treated (iron-overload) immunosuppressed WT (C57BL/6) mice. The data are presented as strip plots with mean ± SD for each group (statistical analysis performed using Mann–Whitney test; significance at *, P ≤ 0.05; **, P ≤ 0.01).

Using our murine OPC model, we first tested whether higher host iron levels lead to an increase in the oral fungal burden during infection. Mice were treated with iron supplement at a dose that increases murine iron levels [19] and iron-treatment was further confirmed by total serum iron measurements in treated and control mice (Figure S1). High iron mice showed significantly higher colony forming units (Cfu)/g of tongue tissue for CAI4 (Ura^+^) *C. albicans*, as compared to untreated control mice (Figure 1). Next, we hypothesized that high iron-mediated increased pathogen level in the oral cavity will cause greater dissemination of *C. albicans* to downstream organs. Indeed, compared to control mice, iron-treated mice with OPC had significantly higher (Cfu)/g of tissue, both in the stomach and the intestine (Figure 1), respectively. This suggests that OPC in higher iron mice is more severe and can lead to a significant increase in the presence of *C. albicans* in the gastrointestinal tract.

### *Changes in iron levels alter* C. albicans *susceptibility to CWP antifungals*

In line with the above results (Figure 1), reduced iron levels in mice with the iron chelator DFX, on the other hand, decreased the oral fungal burden during OPC (Puri et al. 2019b). Furthermore, iron affected the CAI4 (Ura^+^) *C. albicans* cell wall by altering its susceptibility to CWP antifungals (tunicamycin (inhibitor of the synthesis of N-linked oligosaccharide chains on mannoproteins), zymolyase (inhibitor of β-1,3 glucan synthase), or CFW; a chitin-binding dye), *in vitro* [16]. Hence, we next performed CWP antifungal-susceptibility testing on CAI4 (Ura^+^) *C. albicans* isolated from untreated (control) and DFX-treated mice (representing relatively higher and lower host iron environments, respectively) with OPC. Iron-treatment was confirmed by total serum iron measurements in treated and control mice (Figure S1). Homogenized tongue tissue of infected mice was plated on YPD medium alone and YPD containing either tunicamycin, caspofungin (clinical inhibitor of β-1,3 glucan synthase), or CFW.

**Figure 2.**
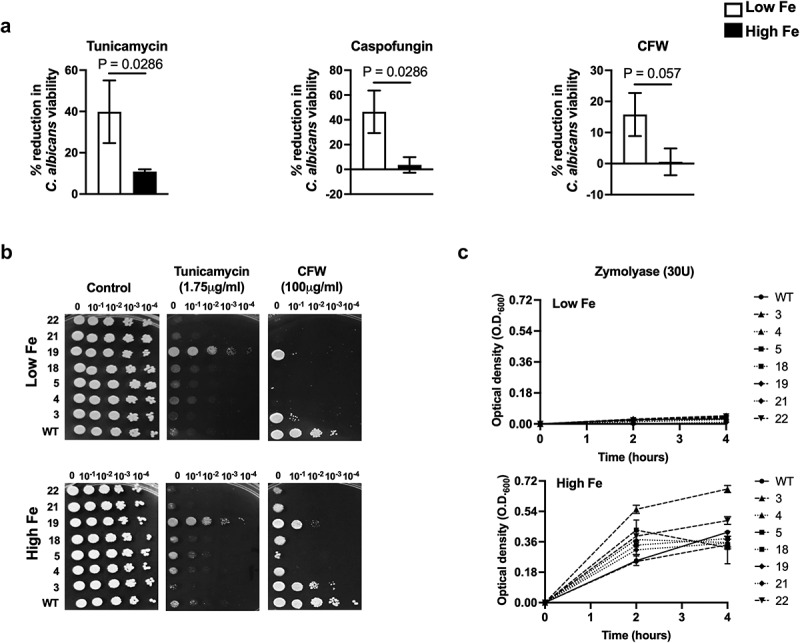
Iron influences antifungal resistance. **(A)** Resistance of CAI4 (Ura^+^) *C. albicans* cells isolated from WT C57BL/6 mice (untreated controls and deferasirox (DFX)-treated (with lower iron levels) immunosuppressed mice) to tunicamycin (1 μg/ml), caspofungin (75 ng/ml) and CFW (12.5 μg/ml) *in vitro*. Viable cell counts were determined by Cfu enumeration over 48 h at 30°C. Sensitivity results are expressed as percent reduction in Cfu on plates containing antifungal agents, compared to growth on control plates lacking any antifungal agent; with statistical comparison of these percent reductions for *C. albicans* isolated from control and DFX-treated mice groups. The data are presented as strip plots with mean ± SD for each group (statistical analysis performed using Mann–Whitney test; significance at *, P ≤ 0.05). (b and c) Resistance of WT SC5314 and oral isolates of *C. albicans* grown in low or high iron medium to CWP agents on respective YNB based media (for 1.75 μg/ml tunicamycin or 100 μg/ml CFW) by spot dilution assay on agar plates after 48 h at 30°C or on liquid media (for 30 U zymolyase) by measuring growth (O.D._600_) for 4 h at 30°C.

All three antifungals caused an increase in viability (3.7-, 12.9-, and 27.8-fold for tunicamycin, caspofungin, and CFW, respectively) for *C. albicans* cells isolated from control mice with higher iron levels, compared to fungal cells isolated from DFX-treated mice with lower iron levels (Figure 2(a)). Tunicamycin and caspofungin results were statistically significant (p = 0.028 for both), while CFW result was close to significance (p = 0.057). This suggested that higher iron levels in the host made *C. albicans* cells more resistant to CWP antifungals. We defined this phenomenon of high iron-mediated resistance to CWP antifungals as the ‘iron-effect’ on antifungal susceptibility.

To examine whether growth in high iron environment similarly affects the CWP antifungal-susceptibility of oral isolates of *C. albicans* as well, we compared the drug sensitivities of *C. albicans* oral isolates grown in low and high iron medium. As observed previously for CAI4 (Ura^+^) *C. albicans* grown *in vitro* [16] or *in vivo* (Figure 1), the WT SC5314 strain grown *in vitro* in high iron was more resistant to tunicamycin and CFW in a spot assay (Figure 2(b)); and showed significantly higher growth in the presence of zymolyase in liquid medium, compared to cells grown in low iron (Figure 2(c)). Almost all of the oral isolates showed this iron-effect, although the actual growth differences in tunicamycin under low and high iron were notably minimal, with one strain (strain-19) showing no iron-effect (Figure 2(b)-middle). In contrast, all oral isolates showed a robust iron-effect when exposed to CFW (Figure 2(b)-right) or zymolyase (Figure 2(c)); with greater resistance in high iron cells, as compared to respective low iron cells. Taken together, a high iron environment makes *C. albicans* cells more resistant to CWP antifungals, both *in vivo* and *in vitro*; and the iron-effect, previously observed for CAI4 (Ura^+^) is largely retained by oral *C. albicans* isolates, especially for antifungals that target the inner cell wall.

### *High iron increases* C. albicans *resistance to commonly used clinical antifungals*

To determine if the iron-effect extends to clinical antifungals, we tested the sensitivities of specific CWP antifungals (caspofungin) and cell membrane-targeting antifungals (amphotericin B, nystatin, and fluconazole) against WT SC5314 *C. albicans* and our oral isolates, under low and high iron (Table 1). Growth in high iron enhanced the caspofungin MIC (1.6 to 8.3-fold increase) for all of the oral isolates, compared to the low iron cells. MIC of high iron cells against amphotericin B increased by 2- to 4-fold, and against nystatin by 2-fold, for six out of the seven isolates tested, as compared to the respective low iron cells. However, no differences in the MIC of fluconazole were observed for cells grown in low or high iron conditions. Our results show that a high iron environment can enhance the antifungal-resistance against a majority of the commonly used clinical antifungals, against *C. albicans* oral isolates.

**Table 1.** Minimum inhibitory concentrations (MIC) of clinical antifungal drugs for oral isolates of *C. albicans.*

Strains	Amphotericin B		Fluconazole		Nystatin		Caspofungin	
	Low Fe medium	High Fe medium	Low Fe medium	High Fe medium	Low Fe medium	High Fe medium	Low Fe medium	High Fe medium
WT	0.5	**1**	1	1	4	4	<0.3	**0.5**
3	1	**2**	2	2	4	4	0.06	**0.5**
4	0.5	**1**	≥16	≥16	4	**8**	<0.3	**0.5**
5	1	1	2	2	4	**8**	0.06	**0.5**
18	0.5	**2**	2	2	4	**8**	<0.3	**0.5**
19	0.5	**2**	1	1	4	**8**	<0.03	**0.25**
21	1	**2**	1	1	4	**8**	<0.03	**0.125**
22	0.5	**2**	2	2	4	**8**	<0.3	**0.5**

Bold numbers represent higher MIC values in high Fe medium as compared to low Fe medium.

### *Levels of* C. albicans *cell wall components of oral isolates show variable response to iron*

**Figure 3.**
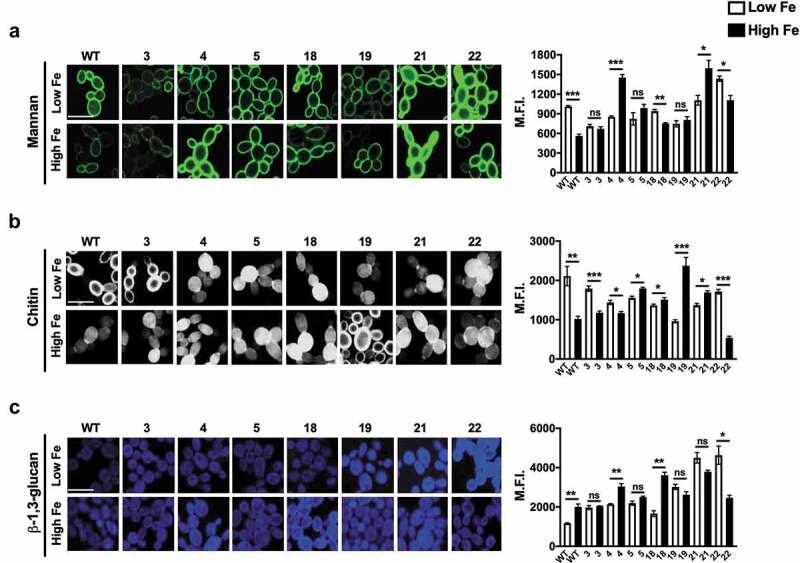
Impact of iron on cell wall architecture of oral isolates. Fluorescent micrographs for WT SC5314 and oral isolates of *C. albicans*, grown in low or high iron medium, stained for mannan (Con A) (a), chitin (CFW) (b), and β-1,3-glucan (AB) (c). Micrographs (scale bar = 5 μm) are representative of three independent replicate experiments. Mean fluorescence intensities (MFI) for n > 150 cells for each growth condition are represented as MFI ± SEM and error bars indicate 95% CI. Data were analyzed using Student’s t-test. *** P ≤ 0.0005; ** P ≤ 0.005; *P < 0.05 and ns = no significance.

Iron-mediated changes in antifungal-susceptibilities towards CWP agents were previously shown to be caused by specific iron-induced alterations in the cell wall architecture of CAI4 (Ura^+^) *C. albicans* [16]. To understand the iron-mediated cell wall changes in oral isolates of *C. albicans*, we compared the levels of mannan, chitin, and β − 1,3 glucan between low and high iron cells of oral isolates of *C. albicans*, along with the WT SC5314 cells. As expected, similar to what was previously reported for CAI4 (Ura^+^) [16], the WT SC5314 strain presented major differences in the levels of cell wall components in response to changes in environmental iron; with high iron cells showing a significant decrease in mannan (Figure 3(a)) and chitin (Figure 3(b)) and a significant increase in β-1,3 glucan (Figure 3(c)), as compared to low iron cells. These changes in specific cell wall components of the WT strain in response to iron were considered as the ‘iron-effect’ for cell wall compositional changes.

With regard to mannan, only 28.57% of the oral isolates showed the iron-effect (with significantly lower mannan levels in high iron cells, compared to cells grown in low iron), while another 28.57% of the isolates showed the opposite effect; 42.86% of the isolates showed no difference between low and high iron cells (Figure 3(a)). For chitin levels, 42.86% of the isolates showed the iron-effect (with significantly lower chitin levels in high iron cells, compared to low iron cells), while the remaining 57.14% isolates showed the opposite effect (Figure 3(b)). Further, β-1,3 glucan levels in cells grown in high iron were significantly higher in 28.57% isolates, compared to low iron cells (thus showing the iron-effect), while 14.29% of the isolates showed the opposite effect; and 57.14% of *C. albicans* isolates showed no difference upon growth in low and high iron (Figure 3(c)). Thus, the effect of iron on the *C. albicans* cell wall composition was not uniform and varied between different oral isolates.

### *High iron increases β-1,3-glucan exposure to enhance phagocytosis of oral* C. albicans *isolates*

**Figure 4.**
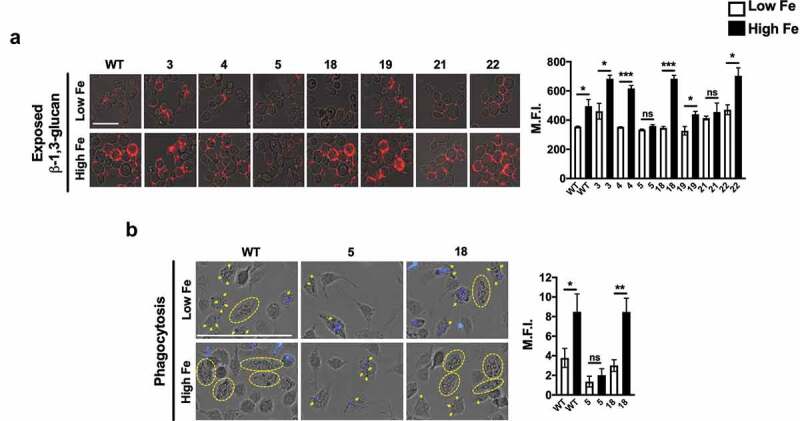
High iron induces enhanced β-1,3-glucan exposure and BMDM-mediated phagocytosis of oral isolates. (a) Fluorescent micrographs for WT SC5314 and oral isolates of *C. albicans*, grown in low or high iron medium, stained with anti-β-1,3-glucan antibody and Cy3-labelled secondary antibody for exposed β-1,3-glucan. Micrographs scale bar = 5 μm. Mean fluorescence intensities (MFI) for n > 150 cells from three independent experiments for each growth condition represented as MFI ± SEM and error bars indicate 95% CI. Data were analyzed using Student’s t test. * P ≤ 0.05; *** P ≤ 0.0005 and ns = no significance. (b) Primary murine-BMDMs were infected with *C. albicans* cells of WT SC5314, and oral isolates 5 and 18, grown in low or high iron medium at MOI of 3 for 1 h at 37°C under 5% CO_2_. Micrographs **(B-left**) showed external *C. albicans* cells stained with CFW (blue color) and phagocytized *C. albicans* cells (unstained; shown by yellow arrows or yellow circles indicate macrophages contained ≥ 6 *C. albicans* cells). Images represent the data derived from three independent experiments in triplicates. Micrographs scale bar = 25 μm. Phagocytic indices were calculated using the following formula: [(total number of engulfed candida/total number of counted macrophages) × (number of macrophages containing engulfed cells/ total number of counted macrophages)] × 100, **(B-right**). The results of triplicates are represented as means ± SEM and error bars indicate 95% CI. Data were analyzed using the Student’s t test. * P ≤ 0.05; ** P ≤ 0.005, and ns = no significance.

We next tested the effect of iron-mediated changes on the incidence of β-1,3-glucan exposure in oral *C. albicans* isolates. To examine this, we compared the levels of β-1,3-glucan exposure in *C. albicans* cells grown in low and high iron. As expected, similar to what we previously reported for CAI4 (Ura^+^) *C. albicans* [16], the WT SC5314 strain showed an increase in β-1,3-glucan exposure upon growth in high iron, compared to low iron cells. Most oral isolates exhibited a similar effect, with a significant increase in β-1,3-glucan exposure levels in high iron, except for two isolates that showed a non-significant increase (Figure 4(a)).

Since exposure of β-1,3-glucan enhances *C. albicans* recognition by immune cells causing an enhanced phagocytic response [16], we next examined if iron-mediated increase in β-1,3-glucan exposure affected macrophage phagocytic-response for our oral isolates. We exposed murine bone-marrow derived macrophages (BMDMs) to low and high iron-grown *C. albicans* WT SC5314 cells and cells of oral isolate 5 and 18 (representing isolates showing non-significant and significant increases in β-1,3-glucan exposure in high iron, respectively). As expected, high iron WT cells showed a significant increase in phagocytosis (2.14-fold increase, compared to low iron cells). Similarly, cells of isolate 5 and 18 grown in high iron also showed an increase in phagocytosis, which was observed to be non-significant (1.48-fold increase) for isolate 5, while significant (2.83-fold increase) for isolate 18 (Figure 4(b)). Thus, high iron caused an increase in β-1,3-glucan exposure among the oral isolates of *C. albicans* tested, and this architectural change has the potential to enhance phagocytosis by macrophages.

## Discussion

The oral cavity as a niche contains a polymicrobial population that has the potential to majorly impact systemic health. High iron modulates the gut microbiome [22] and now we are learning its impact on the oral microbiome. It leads to a more diverse oral microbial community [23] while promoting higher carriage of specific bacteria [24]. Here, we show that iron overload enhances the severity of OPC in mice, as well as the oral-to-gut transmission of *C. albicans* (Figure 1). *Candida* spp. are the fourth leading cause of all microbial bloodstream infections [25]. Contrary to the original belief that catheter lines lead to bloodstream infections and eventual sepsis of *C. albicans*, genotyping studies have confirmed that in the majority of such bloodstream infections, the pathogen originates from the gut [26,27]. Further, it has been reported previously that extensive esophageal candidiasis can lead to gastric candidiasis [28], while we provide here evidence for potential gastrointestinal (GI)-colonization, post-OPC, in our murine model (Figure 1). This suggests that oral *C. albicans* has the potential to cause disseminated disease by modulating fungal levels in the downstream GI tract, specifically in a high iron host.

Besides being the leading cause of OPC, as a key-stone pathogen, *C. albicans* can also cause oral microbial dysbiosis within the host ecosystem and promote aggressive oral infections by encouraging synergistic interactions with oral pathogenic bacteria [29]. Many of these biologically significant interactions involve direct binding of secreted or structural bacterial components to the *C. albicans* cell surface. Niche-specific changes in host iron can alter the *C. albicans* cell wall architecture (Figure 3 [16,30];) and thus hold the potential to alter these inter-kingdom interactions. The Internalin-family protein (InlJ) of the periodontal pathogen *Porphyromonas gingivalis* interacts with the *C. albicans* cell-surface adhesin Als3 that also serves as a ferritin receptor involved in iron uptake and hence regulated by environmental iron [31]. In addition, *C. albicans* MP65, an iron-regulated surface mannoprotein [2] was shown to bind *P. gingivalis* cells. Glucosyltransferase GtfB (an exopolysaccharide) of the caries-causing *Streptococcus mutans* and adhesin SspB (belonging to the AgI/II family polypeptides) of the early colonizer *Streptococcus gordonii* bind to *C. albicans* cell wall mannans or Als3, respectively [32,33]. Also, lack of mannosylated proteins (O- or N-linked mannans) on the *C. albicans* cell wall inhibits its interaction with both these bacteria [32,34]. Iron-mediated changes to the *C. albicans* surface, specifically mannans levels (Figure 3(a) [16,30];) can thus affect periodontal disease and the cariogenic potential in the oral cavity, as well as alter the composition of the early colonizers of the oral cavity [24]. It is, however, imperative to note that clinical strains did not have uniform changes in response to iron. Therefore, both individual’s iron levels and the specific oral strain present can have a major impact on the outcome of various oral diseases.

The non-uniform effect of iron on the cell wall of oral isolates of *C. albicans* can have multiple potential causes. As the outermost layer, mannans face direct exposure of environmental stresses and are greatly influenced by various kinds of oral challenges, such as diverse bacterial microbiota and dietary supplements in the host [35]. Such challenges can cause intrinsic variations in the mannan levels in the cell wall of clinical isolates, prior to those being exposed to high and low iron *in vitro* (Figure 3(a)), thereby causing a variable effect of iron on the mannan content in these isolates. Further, changes in the cell wall mannan can affect the activity of the cell wall associated proteins that participate in cell wall morphogenesis, such as chitinases (hydrolyses chitin), β‐1,3-glucanases (hydrolyses β‐1,3-glucan), and cell wall remodeling enzymes (e.g. β‐1,3-glucan transferase *BGL2*) [36]. These in turn can explain the inter-strain variation in chitin and β‐1,3-glucan levels among the oral isolates (Figure 3(b,c)).

A decrease in mannans, an increase in chitin, and a decrease in β‐1,3-glucan in the WT *C. albicans* cell wall led to higher tunicamycin, CFW, and zymolyase (including caspofungin) resistance, respectively, under high iron (Figure 2(b,c), Table 1), similar to previous observation [16]. Interestingly, despite inter-strain variations in the effect of iron on the cell wall components of *C. albicans* oral isolates, most showed the iron-effect on drug-resistance (Figure 2(b,c), Table 1). The effect of high iron on amphotericin B was more pronounced than that observed for nystatin (Table 1), although both represent polyene antifungals. Structural differences between the two drugs that bestow higher antifungal activity on amphotericin B over nystatin [37] may explain this observation. Interestingly, iron levels had no effect on the fluconazole MIC for any of the strains tested (Table 1). This is potentially a result of the fact that fluconazole treatment of *C. albicans* causes extensive remodeling of iron homeostasis networks [38] that may limit the actual changes in intracellular iron levels caused by growth in low and high iron.

Mitogen activated protein kinase (MAPK) Cek1 activation can impact sensitivity towards tunicamycin and CFW [39,40]; while treatment with β‐1,3-glucan inhibitor (glucanase) enhances the activation of Slt2p (homologous to the *C. albicans* MAPK Mkc1 cell wall integrity pathway) in *Saccharomyces cerevisiae* [41]. Iron is a well-known inducer of Cek1, while MKc1 plays a cooperative role along with Cek1 in the regulation of the cell wall architecture [42]. Thus, iron-induced MAPK signaling as alternative mechanisms for high iron-mediated increase in resistance of WT and oral isolates of *C. albicans* to CWP agents need to be explored with respect to iron.

## Supplementary Material

Supplemental Material
