# Supplementary material for: High iron-mediated increased oral fungal burden, oral-to-gut transmission, and changes to pathogenicity of Candida albicans in oropharyngeal candidiasis
Source: J Oral Microbiol. 2022 Mar 1;14(1):2044110. doi: 10.1080/20002297.2022.2044110 (PMC8896197; doi:10.1080/20002297.2022.2044110)
Supplement: Supplemental Material [file ZJOM_A_2044110_SM9086.docx]

**Supplemental Figure S1.** Estimation of total serum iron in mice. Total serum iron was measured in blood collected from Control, Iron-overload and Deferasirox (DFX) mice. The data are presented as strip plots with mean ± SD for each group (statistical analysis performed using Mann-Whitney test; significance at *, P ≤ 0.05; **, P ≤ 0.01).

**Supplemental Figure S2.** Molecular characterization of oral clinical isolates. PCR amplification of a DNA fragment from clinical isolates using *C. albicans*-specific primers. *C. albicans* CAI4 (Ura+) and WT SC5314 were used as positive controls while non-albicans *Candida* spp. were used as negative controls.

**Supplemental methods:**

Murine serum iron measurements. On day 5 of respective treatments (as detailed in Material and methods), total serum iron levels were measured by a colorimetric assay, using an iron assay kit (#MAK025; Sigma) as per manufacturer’s instructions, in serum prepared from collected mice blood.

Molecular characterization of oral clinical isolates. DNA fragment from clinical isolates and respective controls were PCR-amplified using *C. albicans*-specific primers against the *C. albicans* *EFB1* gene, as described previously (Maneu and Gozalbo, 2000).

References

Maneu V, Martínez JP, Gozalbo D. Identification of Candida albicans clinical isolates by PCR amplification of an EFB1 gene fragment containing an intron-interrupted open reading frame. Med Mycol. 2000 Apr;38(2):123-6. doi: 10.1080/mmy.38.2.123.126. PMID: 10817228.
